# Social Support and Locus of Control in Subclinical Eating-Disorder Tendencies in a Romania-Based Convenience Sample

**DOI:** 10.3390/diseases14080269

**Published:** 2026-07-24

**Authors:** Denisa-Catalina Dragomir, Adelaida-Sorana Trifu, Amelia-Damiana Trifu

**Affiliations:** 1 Faculty of Psychology and Educational Studies, University of Bucharest, 050663 Bucharest, Romania; denisa-catalina.dragomir91@s.fpse.unibuc.ro; 2 Department of General Medicine, Carol Davila University of Medicine and Pharmacy, 020021 Bucharest, Romania; amelia-damiana.trifu0720@stud.umfcd.ro

**Keywords:** subclinical eating-disorder tendencies, body image concerns, perceived social support, locus of control, binge-eating and bulimic tendencies

## Abstract

Background: Subclinical eating-disorder tendencies may reflect early psychological vulnerability before the development of clinically diagnosed eating disorders. Body image concerns, perceived social support, and control-related beliefs are established correlates of eating pathology. However, their joint pattern across several eating-disorder tendency profiles has received less attention in Romanian nonclinical adult samples. Objective: We examined differences in perceived social support, locus of control, and body image concerns among participants with and without subclinical tendencies associated with anorexia, bulimia, and binge eating. We investigated the association between locus of control and body image concerns. Methods: A cross-sectional quantitative design was used. The sample included 108 participants who completed online self-report measures assessing perceived social support, eating-disorder tendencies, body image concerns, and locus of control. Independent-samples *t*-tests and Welch tests were used for group comparisons. Bonferroni correction was applied within families of comparisons. Effect sizes were reported using Hedges’ g. Pearson correlation and simple linear regression were used to examine the association between locus of control and body image concerns. Results: Body image concerns were significantly higher among participants with anorexic, binge-eating, and bulimic tendencies. Perceived social support was lower among participants with binge-eating and bulimic tendencies, but not among those with anorexic tendencies. Locus of control differed significantly only in the exploratory bulimia comparison after correction. External locus of control was positively associated with body image concerns and explained a small but significant proportion of variance. Conclusions: Body image concerns emerged as the most consistent correlate of subclinical eating-disorder tendencies. The findings support the relevance of body image, interpersonal resources, and control-related beliefs in early eating-disorder vulnerability.

## 1. Introduction

Eating disorders are complex psychological conditions influenced by biological, psychological, interpersonal, and sociocultural factors. Recent reviews emphasize that risk factors for eating disorders are multidimensional and include individual vulnerabilities, body-related concerns, interpersonal difficulties, emotional problems, and broader sociocultural pressures [1]. Although clinical eating disorders represent the most severe forms of pathology, subclinical eating-disorder tendencies are also important because they may indicate early psychological vulnerability and may precede the development of more severe symptoms. Therefore, examining psychological factors associated with subclinical eating-disorder tendencies may contribute to prevention and early identification. Research on eating-disordered behavior has long extended beyond formally diagnosed clinical populations. The Eating Attitudes Test was introduced in the late 1970s as a measure of eating-disorder symptomatology, and early studies subsequently applied screening methods in student and community populations. For example, Button and Whitehouse used the term “subclinical anorexia nervosa” in their 1981 investigation of college students [2]. Later scholarship conceptualized disturbed eating along a spectrum ranging from dieting, weight and shape preoccupation, and subthreshold symptoms to partial and full-syndrome eating disorders [3]. Consequently, subclinical eating-disordered behavior should be understood as a longstanding area of research rather than as a recently identified phenomenon. The present study is situated within this broader tradition while focusing specifically on the joint pattern of body image concerns, perceived social support, and locus of control in a Romanian sample.

One of the most consistently studied factors in eating-disorder research is body image disturbance. Body image is a multidimensional construct that includes perceptual, cognitive, affective, and behavioral components. Prnjak emphasized that body image disturbance is central to the psychopathology of eating disorders and that different dimensions of body image may be differently related to eating-disorder outcomes [4]. In a prospective study, researchers also found that specific aspects of body image, such as weight and shape dissatisfaction, overvaluation of weight and shape, preoccupation with weight and shape, and fear of weight gain, may have distinct roles in the onset and maintenance of eating-disorder symptoms [5]. These findings support the idea that body image concerns should be considered a key variable when examining vulnerability to anorexic, bulimic, and binge-eating tendencies.

Additional empirical studies also support the central role of body image concerns in disordered eating. Aparicio-Martinez et al., in a study of college women, found that body dissatisfaction, body concerns, and the desire to achieve a thinner body image were associated with disordered eating attitudes [6]. Similarly, Mallaram, in a study of female medical students, showed that body image perception was related to eating-disorder behavior, perceived stress, self-esteem, and quality of life [7]. These findings are important because they show that body image concerns are relevant not only in clinical populations, but also among students and young adults who may present subclinical or emerging symptoms.

The relevance of body image is not limited to restrictive eating pathology. Research on binge-eating disorder also suggests that body image disturbance is an important feature of binge-eating symptoms. In a review of body image disturbance in binge-eating disorder, Lewer et al. showed that individuals with binge-eating disorder frequently present cognitive-affective disturbances related to body image, including dissatisfaction and overvaluation of shape and weight [8]. Similarly, Bianchi found that body image impact on quality of life was associated with adolescents’ binge eating, and that body image coping strategies played an indirect role in this relationship [9]. These findings indicate that body image concerns may function as a transdiagnostic factor across different types of eating-disorder tendencies.

Sociocultural influences on body image and disordered eating have been studied for several decades [10,11]. Earlier research described widespread dissatisfaction with weight and appearance and examined how cultural standards, interpersonal comparison, and exposure to idealized bodies may contribute to eating-related concerns [10,12]. Studies conducted before the emergence of contemporary social networking platforms linked exposure to thin-ideal media and internalization of appearance ideals with body dissatisfaction and eating-disorder symptoms, and experimental findings were subsequently synthesized in meta-analytic research [11,13]. Contemporary social media should therefore be viewed as a newer context through which established sociocultural mechanisms—such as ideal internalization, appearance comparison, and perceived pressure to be thin—may operate [14,15]. Social networking platforms may add distinctive features, including highly visual content, algorithmic personalization, interactivity, and continuous exposure, but the broader relationship between media ideals, body dissatisfaction, and disordered eating is not new [14,15,16,17].

Building on this earlier mass-media literature, recent studies have examined whether image-based and interactive social media environments intensify or modify these established processes. In a systematic review, Holland and Tiggemann concluded that social networking site use is associated with body image and disordered-eating outcomes [15]. Aparicio-Martinez further emphasized that social media may contribute to the internalization of unrealistic beauty ideals, body dissatisfaction, and disordered eating attitudes [6]. Sanzari et al. compared demographically diverse undergraduate student cohorts surveyed in 2015 and 2022. Participants in the 2022 cohort reported greater body image disturbance, more frequent vomiting and laxative use, and greater engagement with multiple and image-based social media platforms [18]. Together, these findings indicate that established sociocultural appearance pressures may also operate through contemporary social media environments.

Another important variable in eating-disorder research is perceived social support. Social support may influence eating-related symptoms by providing emotional validation, interpersonal security, and adaptive coping resources. In an integrative review, the authors emphasized the relevance of social support networks in eating disorders, while also noting that the literature on this topic remained less consolidated than other areas of eating-disorder research [19]. More recent findings support the importance of social support in eating-disorder symptoms. Kim found deficits in subjective social support across eating-disorder diagnostic groups, suggesting that individuals with eating disorders may perceive their interpersonal environment as less supportive [20]. In addition, Makri found that patients with eating disorders reported higher loneliness and lower perceived social support compared to healthy controls [21].

Longitudinal research further suggests that the relationship between social support and eating-disorder symptoms may be reciprocal. Stern found prospective reciprocal associations between perceived peer support and eating-disorder symptoms in adolescent girls [22]. Specifically, lower perceived peer support predicted later eating-disorder symptoms, while higher eating-disorder symptoms also predicted later reductions in perceived peer support. Cortés-García also showed prospective associations between loneliness and disordered eating from early adolescence to adulthood, further supporting the importance of interpersonal factors in eating-disorder vulnerability [23]. Together, these findings suggest that interpersonal difficulties may contribute to eating-disorder symptoms, while eating-disorder symptoms may also reduce perceived social connectedness.

Compared with body image and social support, locus of control has been less frequently examined in relation to eating-disorder tendencies, but it remains theoretically relevant. Locus of control refers to the extent to which individuals perceive outcomes as being determined by their own actions or by external forces. In the context of eating-related vulnerability, a more external locus of control may be associated with feelings of helplessness, reduced perceived agency, and greater dependence on external evaluation. Di Corrado et al. found that internal locus of control and self-efficacy were associated with body image outcomes in a path-analysis model [24], whereas Froreich et al. linked broader control-related dimensions to disordered-eating behaviors [25]. These findings provide indirect theoretical context, but neither study directly tested generalized external locus of control in participants with the study-specific subclinical anorexia classification. The present uncorrected anorexia-related finding should therefore be treated as tentative.

The broader literature also suggests that eating-disorder vulnerability rarely results from a single factor. For example, Manaf et al. found relationships among negative body image, depression, and susceptibility to eating disorders in female medical undergraduate students, with depression partially mediating the relationship between body image and eating-disorder susceptibility [26]. More recent network-analysis research among college students with subclinical eating-disorder symptoms identified preoccupation with food and eating and weight/shape dissatisfaction as influential symptoms, while difficulties in emotional clarity and non-acceptance of emotions acted as bridge nodes linking emotion dysregulation with eating-disorder symptoms [27]. These findings support the examination of eating-disorder tendencies through models that include body-related, emotional, interpersonal, and control-related dimensions.

The previous studies indicate that body image concerns, perceived social support, and control-related beliefs are relevant in the study of eating-disorder symptoms. Substantial research has examined eating-disorder symptoms, body dissatisfaction, and disordered eating in both clinical and nonclinical populations [3,4,28]. Most evidence concerning the psychosocial correlates of eating-disorder symptoms has been generated in North American and Western European samples. Available evidence from Romania and Central and Eastern Europe indicates that eating-disorder risk and body image concerns are also relevant in this region, although findings concerning cultural differences are not uniform [29,30]. Cross-European research involving Romanian university students has identified broadly similar eating-disorder screening patterns across several European countries, whereas a direct comparison of Romanian and German students found higher Romanian scores on several Eating Disorder Inventory dimensions [31]. Differences have also been reported among cultural groups within Romania. Thus, a simple Western–Eastern European distinction may be inadequate. Therefore, the present gap does not indicate an absence of research on subclinical eating-disordered behavior [3,28,32]. Body image concerns, perceived social support, and locus of control have each been examined in relation to eating pathology, but largely in separate or only partially overlapping lines of research [4,19,24,25,32]. To our knowledge, comparatively few studies have examined all three variables simultaneously across anorexic, binge-eating, and bulimic tendency profiles. This particular combination appears especially underexamined in heterogeneous Romanian adult samples, as available Romanian research has generally focused on adolescents, university students, or specific occupational groups [33,34,35]. The present research addresses this narrower question by comparing participants with and without anorexic, binge-eating, and bulimic tendencies on these three psychological dimensions.

The main objective of the present research was to examine whether perceived social support, locus of control, and body image concerns differ according to the presence or absence of subclinical eating-disorder tendencies. Specifically, the study focused on anorexic tendencies, bulimic tendencies, and binge-eating tendencies. A secondary objective was to examine the association between locus of control and body image concerns.

Based on these objectives, the following hypotheses were formulated:

**H1.**  *Participants with anorexic tendencies will report greater body image concerns, lower perceived social support, and a more external locus of control than participants without such tendencies.*

**H2.**  *Participants with binge-eating tendencies will report greater body image concerns, lower perceived social support, and a more external locus of control than participants without such tendencies.*

**H3.**  *Participants with bulimic tendencies will report greater body image concerns, lower perceived social support, and a more external locus of control than participants without such tendencies.*

**H4.**  *A more external locus of control will be positively associated with body image concerns.*

Body image concerns and interpersonal factors have long been studied in relation to eating pathology, whereas general locus of control has less often been incorporated into the same empirical model. The Romanian context also provides locally relevant evidence from a setting in which population-based research remains more limited than in several Western European and North American contexts. The findings should therefore be interpreted as a regional and integrative extension of an established literature, rather than as the first substantial investigation of subclinical eating-disordered behavior [33,34].

## 2. Materials and Methods

A cross-sectional online survey was conducted with a convenience sample of adults. Participants completed self-report measures of eating-disorder tendencies, perceived social support, body image concerns, and locus of control. Participants were informed about the purpose of the study, the anonymous and confidential processing of their responses, and their right to withdraw at any time without consequences. Participation was voluntary and not financially rewarded. The estimated completion time was approximately 10–15 min.

The final sample consisted of 108 participants and was predominantly female. Ninety-four participants were female (87.0%) and 14 were male (13.0%). Regarding area of residence, 58 participants were from urban areas (53.7%) and 50 were from rural areas (46.3%). In terms of occupation, the sample included pupils, students, employed participants, participants who were both students and employed, and participants without stable employment. The educational level of the participants varied from lower secondary education to postgraduate study. These demographic characteristics indicate that the sample was heterogeneous in terms of education and occupational status, but unbalanced in terms of gender distribution.

No formal a priori power analysis was conducted. Recruitment used a convenience-sampling approach, and all eligible participants who completed the questionnaire during the study recruitment period were considered for inclusion. Because the eating-disorder tendency groups were created after the EDDS responses had been scored, the number of participants meeting each tendency criterion could not be predetermined. The final sample comprised 108 participants, including 17 participants who met the study-specific bulimic-tendency criterion and 91 who did not.

An approximate sensitivity analysis was conducted for the bulimia-related independent-group comparisons. With group sizes of 17 and 91, a two-sided significance level of α = 0.0167—corresponding to the Bonferroni-adjusted threshold for the three outcomes—and 80% statistical power, the available sample was capable of detecting standardized mean differences of approximately *d* = 0.87 or larger. At the conventional unadjusted level of α = 0.05, the corresponding detectable difference was approximately *d* = 0.75. Therefore, the bulimia-related analyses had adequate sensitivity primarily for large group differences and limited sensitivity for small or moderate differences; these analyses were consequently treated as exploratory.

Perceived social support was measured using the Social Provisions Scale (SPS), originally developed by Russell and Cutrona [36]. The scale contains 24 items, including both directly scored and reverse-scored items. Responses are provided on a four-point Likert scale, ranging from disagreement to agreement. The validated Romanian-language version was administered [37]. After reverse-scoring the negatively keyed items, a total score was calculated, with higher scores indicating greater perceived social support. In the present sample, the 24-item total score showed excellent internal consistency (Cronbach’s α = 0.92).

The instrument assesses several dimensions of perceived social support, including attachment, social integration, reassurance of worth, reliable alliance, guidance, and opportunity for nurturance. A total score was calculated by summing the relevant items after applying the required reverse scoring. Higher total scores indicate a higher level of perceived social support.

Eating-disorder tendencies were assessed using the Eating Disorder Diagnostic Scale [38], administered as an online self-report questionnaire. The instrument was used to assess participants’ self-reported symptoms associated with anorexia, bulimia, and binge eating. No clinical interview, physical examination, or clinician-established diagnosis was conducted. Consequently, the classifications described below represent study-specific indicators of self-reported symptom tendencies and should not be interpreted as clinical diagnoses.

For descriptive purposes, three continuous symptom-component scores were calculated. The anorexia-related score was obtained by summing EDDS Items 2, 3, and 4. The bulimia-related score was obtained by summing Items 5, 6, and 8. The binge-eating-related score was obtained by summing Items 5, 6, 7, and 9–14.

For the group comparisons, binary self-report classifications were created according to predefined operational rules. Participants were classified as presenting anorexia-related tendencies when the sum of EDDS Items 2–4 was at least 12 or when their BMI was below 17.5 kg/m^2^. Internal consistency was calculated for the three continuous EDDS-derived symptom scores reported. Cronbach’s alpha was 0.86 for the anorexia-related score comprising Items 2–4, 0.46 for the bulimia-related score comprising Items 5, 6, and 8, and 0.84 for the binge-eating-related score comprising Items 5–7 and 9–14. These coefficients refer to the continuous summed scores and not to the dichotomous tendency classifications, which were generated using the operational decision rules described below. Participants were classified as presenting bulimia-related tendencies when the sum of Items 5, 6, and 8 was at least 4, the sum of compensatory-behavior Items 15–18 was at least 8, and either Item 3 or Item 4 had a score of at least 4. Participants were classified as presenting binge-eating-related tendencies when the sum of Items 5, 6, and 7 was at least 4.

The three classifications were calculated independently and were therefore not mutually exclusive. Meeting one of these operational criteria indicates an elevated self-reported tendency within the present study and does not establish the presence of anorexia nervosa, bulimia nervosa, binge-eating disorder, or any other clinical condition. The low internal consistency of the three-item bulimia-related continuous score indicates that its components should not be interpreted as a strongly homogeneous scale. Accordingly, findings involving this score and the corresponding exploratory classification were interpreted cautiously.

BMI was calculated from participants’ self-reported weight and height and was therefore also based on self-report information.

Body image concerns were measured using the Body Shape Questionnaire—BSQ-16B, a shortened version of the Body Shape Questionnaire [39,40]. The instrument contains 16 items and uses Likert-type response options. The scale assesses concerns related to body shape, body dissatisfaction, and preoccupation with physical appearance.

A total score was calculated by summing the responses to the items. Higher scores indicate a higher level of concern or dissatisfaction regarding body shape. The BSQ-16B total score showed excellent internal consistency in the present sample (Cronbach’s α = 0.96). Therefore, in the present study, the variable was interpreted as body image concern or body shape dissatisfaction, rather than as positive body image.

Locus of control was assessed using Rotter’s Locus of Control Scale [41]. The instrument consists of paired statements, and participants select the option that best reflects their personal beliefs. The scale measures the extent to which individuals perceive life outcomes as being determined by their own actions or by external factors such as chance, fate, or the influence of other people.

A total score was calculated according to the instrument’s scoring procedure. Six filler items were excluded from scoring, and the total was based on the 23 scored dichotomous items. Higher scores indicate a more external locus of control, whereas lower scores indicate a more internal locus of control. Internal consistency in the present sample was modest (KR-20 = 0.61).

Except the SPS, the other scales were translated into Romanian using forward translation, independent back-translation, reconciliation, and pilot testing. However, a full Romanian psychometric validation and cross-cultural measurement-invariance analysis were not conducted.

After data collection, the database was screened before analysis. The screening process included checking the completeness of responses, identifying extreme values, verifying the coding of variables, and preparing the total scores and grouping variables needed for the statistical procedures.

Data were organized in SPSS v27.0 format and analyzed using standard procedures for descriptive statistics, independent-samples comparisons, correlation, and regression.

The statistical analyses were planned according to the objectives and hypotheses of the study. First, descriptive statistics were calculated for the main continuous variables, including perceived social support, body image concerns, locus of control, anorexic tendencies, bulimic tendencies, and binge-eating tendencies. The descriptive analysis included indicators such as mean, standard deviation, minimum and maximum values, skewness, and kurtosis.

Before conducting inferential analyses, the assumptions required for parametric tests were examined. The distribution of the variables was evaluated using skewness and kurtosis indicators. The presence of extreme values was also checked. For group comparisons, homogeneity of variance was assessed using Levene’s test with a significance threshold of *p* < 0.05. For the anorexia- and binge-eating-related comparisons, the standard independent-samples Student’s *t*-test was reported when Levene’s test was non-significant (*p* ≥ 0.05), whereas Welch’s *t*-test was reported when Levene’s test was significant (*p* < 0.05). For the exploratory bulimia-related comparisons, Welch’s *t*-test was used for all outcomes because the groups were markedly unequal in size (91 versus 17 participants), irrespective of the Levene’s test result. The corresponding Levene statistics are reported with each set of comparisons. Welch’s correction or non-parametric alternatives, such as the Mann–Whitney U test, were considered appropriate options depending on the distribution of the variables and the equality of variances.

To examine the association between locus of control and body image concerns, Pearson correlation analysis was planned. If the assumptions for Pearson correlation were not adequately met, Spearman correlation was considered as an additional non-parametric alternative.

Because multiple group comparisons were conducted within each eating-disorder tendency category, Bonferroni correction was applied separately for each family of comparisons. Since three dependent variables were compared within each category, the adjusted significance threshold was set at *p* < 0.017. For group comparisons, effect sizes were calculated using Hedges’ g, with 95% confidence intervals, in order to evaluate the magnitude and precision of the differences between groups. Hedges’ g was preferred because some group sizes were unequal.

In addition to the primary hypothesis-driven analyses, exploratory demographic analyses were conducted according to age group and gender. Differences among the four age categories were examined using Welch’s one-way analysis of variance because the age groups were unequal in size. Gender differences were examined using Welch’s independent-samples *t*-test because the male and female groups were substantially unequal. Perceived social support, locus of control, body image concerns, and BMI were examined as outcomes. Because four outcomes were evaluated within each demographic analysis, a Bonferroni-adjusted significance threshold of *p* < 0.0125 was used. These analyses were considered secondary and exploratory.

A simple linear regression analysis was planned to examine whether locus of control was statistically associated with body image concerns when body image concern was treated as the criterion variable. In this model, locus of control was entered as the predictor variable and body image concern as the dependent variable. The regression analysis was used only to examine statistical association and explain variance, not to establish causality.

As secondary analyses, the continuous EDDS-derived anorexia-related, binge-eating-related, and bulimia-related symptom-component scores were examined. Spearman rank-order correlations were calculated between each continuous symptom-component score and perceived social support, locus of control, and body image concerns. Bias-corrected and accelerated 95% bootstrap confidence intervals were estimated using 10,000 resamples. Holm correction was applied across the nine correlations. Three separate multiple linear regression models were then estimated, with the anorexia-related, binge-eating-related, and bulimia-related continuous scores entered separately as dependent variables and perceived social support, locus of control, and body image concerns entered simultaneously as predictors. HC3 heteroskedasticity-robust standard errors were used. Unstandardized and standardized coefficients, 95% confidence intervals, adjusted R^2^ values, robust model F tests, and variance-inflation factors were reported. Holm-adjusted *p* values across the nine regression coefficients were also calculated as a conservative multiple-testing sensitivity analysis. Because of the low internal consistency of the three-item bulimia-related score, analyses involving this score were considered exploratory.

## 3. Results

The main variables included in the analyses were perceived social support, body image concerns, locus of control, anorexic tendencies, bulimic tendencies, and binge-eating tendencies. Descriptive indicators, including mean, standard deviation, minimum and maximum values, skewness, and kurtosis, were examined before conducting inferential tests. The values of skewness and kurtosis were considered acceptable for the use of parametric analyses. However, for group comparisons, the assumption of homogeneity of variance was also examined separately using Levene’s test.

The final sample consisted of 108 participants. The majority of participants were female. Regarding area of residence, slightly more than half of the participants came from an urban environment. The sample included participants with different occupational statuses and educational levels (Table 1).

Descriptive statistics were calculated for the main continuous variables included in the study. These variables were perceived social support, body image concerns, locus of control, anorexic tendencies, bulimic tendencies, and binge-eating tendencies. The results are presented in Table 2.

The descriptive indicators showed that the distributions of the main variables were generally acceptable for parametric analysis. Perceived social support showed a moderate negative skewness, suggesting that many participants reported relatively high levels of perceived social support. Body image concerns and binge-eating tendencies showed positive skewness, indicating that higher scores were less frequent. Bulimic tendencies also showed positive skewness, suggesting that most participants obtained lower scores on this variable.

Participants were classified into groups according to the presence or absence of specific eating-disorder tendencies. Separate grouping variables were used for anorexic tendencies, bulimic tendencies, and binge-eating tendencies. The distribution of participants across these groups is presented in Table 3. The classifications reported in Table 3 were based exclusively on participants’ self-reported EDDS responses and, for the anorexia-related classification, self-reported anthropometric information. They represent operational indicators of elevated symptom tendencies rather than clinical diagnoses. Because each criterion was calculated independently, participants could meet more than one classification.

The group distribution indicated that the anorexia-related and binge-eating-related groups were sufficiently represented for independent-samples comparisons. However, the bulimia-related groups were more unequal in size, with a relatively small number of participants classified as presenting bulimic tendencies. Therefore, comparisons involving bulimic tendencies should be interpreted with caution and, where necessary, tested using statistical procedures robust to unequal variances and unequal group sizes.

Cross-tabulation of the three eating-disorder tendency classifications indicated substantial overlap between the groups. Of the 108 participants, 55 did not meet any of the three tendency classifications, 28 met exactly one classification, 10 met exactly two classifications, and 15 met all three classifications. Thus, 25 participants, representing 23.1% of the total sample and 47.2% of participants with at least one elevated tendency, met criteria for two or more tendency classifications. Overlap was particularly pronounced for bulimic tendencies: all 17 participants classified with bulimic tendencies were also classified with binge-eating tendencies, and 15 of these participants were additionally classified with anorexic tendencies. The tendency groups should therefore be understood as overlapping screening classifications rather than mutually exclusive profiles.

The first hypothesis examined whether participants with anorexic tendencies differed from participants without anorexic tendencies in perceived social support, locus of control, and body image concerns. Independent-samples comparisons were conducted for the three dependent variables. For body image concerns, Welch’s correction was used because the assumption of equal variances was not met. The results are presented in Table 4. Levene’s test was nonsignificant for perceived social support, *F*(1, 106) = 1.82, *p* = 0.180, and locus of control, *F*(1, 106) = 1.00, *p* = 0.320; therefore, Student’s *t*-tests were reported for these outcomes. Levene’s test was significant for body image concerns, *F*(1, 106) = 30.97, *p* < 0.001; therefore, Welch’s *t*-test was reported.

The results showed no significant difference between participants with and without anorexic tendencies in perceived social support. The difference in locus of control was statistically significant at the uncorrected level, with a small-to-moderate effect size; however, this result did not remain significant after Bonferroni correction. The difference in body image concerns was statistically significant and showed a large effect size, indicating that participants with anorexic tendencies reported substantially higher concern or dissatisfaction regarding body shape. Therefore, the first hypothesis was partially supported.

The second hypothesis examined whether participants with binge-eating tendencies differed from participants without binge-eating tendencies in perceived social support, locus of control, and body image concerns. Independent-samples comparisons were conducted for the three dependent variables. When the assumption of homogeneity of variance was not met, Welch’s correction was used. The results are presented in Table 5. Levene’s test was significant for perceived social support, *F*(1, 106) = 4.50, *p* = 0.036, and body image concerns, *F*(1, 106) = 9.24, *p* = 0.003; therefore, Welch’s *t*-tests were reported for these outcomes. Levene’s test was nonsignificant for locus of control, *F*(1, 106) = 0.58, *p* = 0.447; therefore, the standard Student’s *t*-test was reported.

The results showed that participants with binge-eating tendencies reported significantly lower perceived social support than participants without binge-eating tendencies, with a medium effect size. No statistically significant difference was found between the two groups regarding locus of control. Participants with binge-eating tendencies reported significantly higher body image concerns than participants without binge-eating tendencies, with a large effect size. After Bonferroni correction, the differences in perceived social support and body image concerns remained statistically significant. Therefore, the second hypothesis was partially supported.

Exploratory comparisons were conducted for the bulimia-related groups. Levene’s tests were nonsignificant for perceived social support, *F*(1, 106) = 2.23, *p* = 0.138, locus of control, *F*(1, 106) = 0.09, *p* = 0.765, and body image concerns, *F*(1, 106) = 0.22, *p* = 0.642. Nevertheless, Welch’s *t*-test was used for all three comparisons because the groups were markedly unequal in size (91 participants without and 17 participants with bulimic tendencies), and these analyses were considered exploratory. The results are presented in Table 6.

Exploratory Welch tests showed that participants with bulimic tendencies reported lower perceived social support, t(20.44) = 2.62, *p* = 0.016, Hedges’ g = 0.77; a more external locus of control, t(25.26) = −3.08, *p* = 0.005, Hedges’ g = −0.72; and substantially higher body image concerns, t(22.58) = −7.31, *p* < 0.001, Hedges’ g = −1.90. All three comparisons met the within-family Bonferroni-adjusted threshold of *p* < 0.0167, although the social-support result was close to the threshold and should be interpreted cautiously.

Overall, the group comparisons showed that body image concerns differed consistently according to eating-disorder tendency status. Participants with anorexic tendencies, binge-eating tendencies, and bulimic tendencies obtained higher scores on body image concerns than participants without these tendencies. Perceived social support differed significantly in the comparisons involving binge-eating and bulimic tendencies, with participants presenting these tendencies reporting lower perceived social support. However, perceived social support did not differ significantly according to anorexic tendency status.

**Figure 1:**
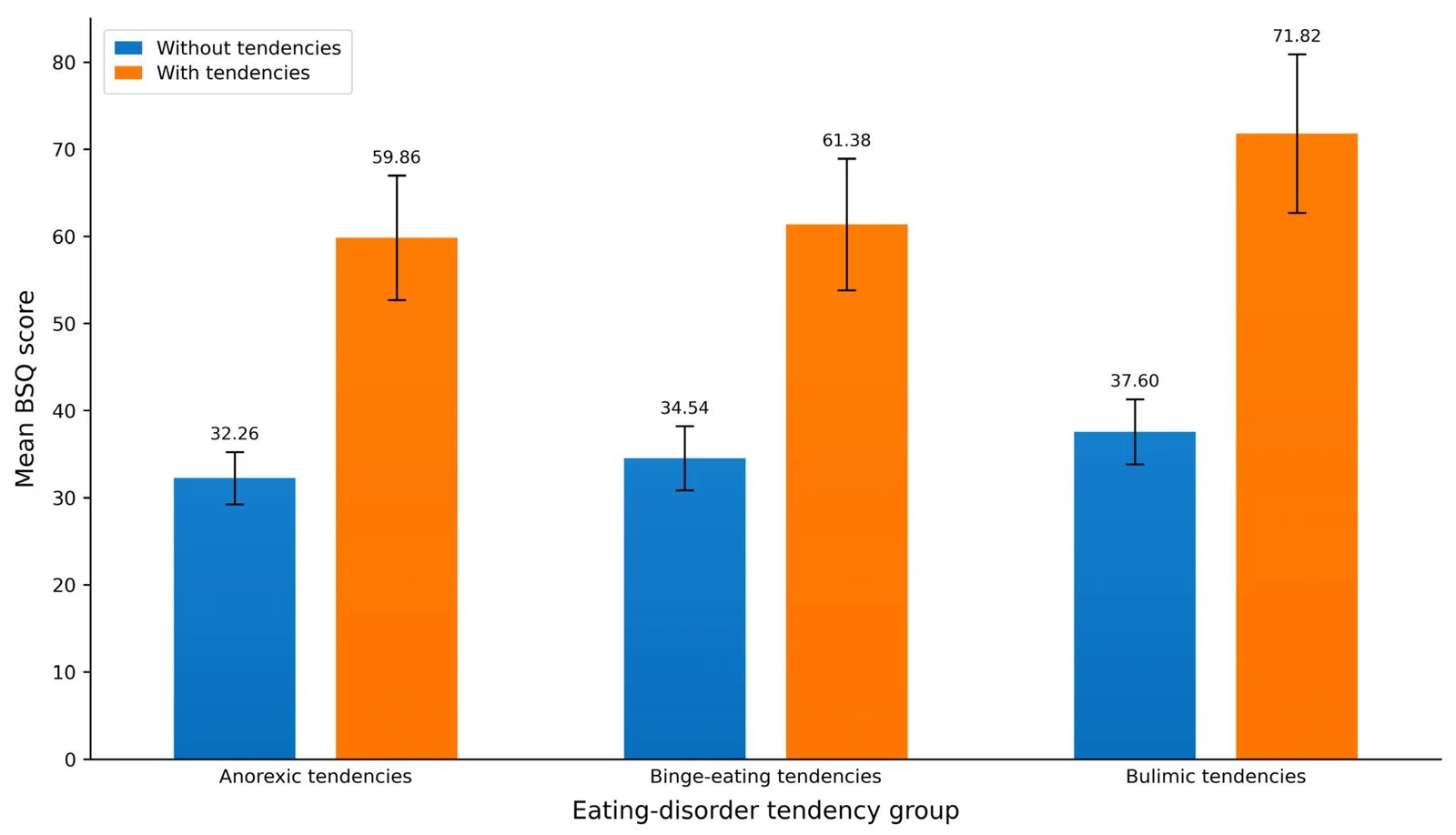
Mean body image concern scores according to eating-disorder tendency status. Error bars represent 95% confidence intervals for the group means. Higher BSQ scores indicate greater concern or dissatisfaction regarding body shape. The anorexic-, binge-eating-, and bulimic-tendency classifications were analyzed separately and were not mutually exclusive.

To provide a clearer visual representation of the group differences, Figure 1 presents the mean body image concern scores and their 95% confidence intervals for participants with and without anorexic, binge-eating, and bulimic tendencies. Across all three classifications, participants meeting the tendency criteria obtained higher mean BSQ scores than participants not meeting the corresponding criteria. Higher BSQ scores indicate greater concern or dissatisfaction regarding body shape.

At the uncorrected level, locus of control differed between participants with and without anorexic tendencies; however, this result did not remain significant after Bonferroni correction. After correction, locus of control differed significantly only in the exploratory bulimia comparison. No significant difference was found for binge-eating tendencies.

To examine the association between locus of control and body image concerns, a Pearson correlation analysis was conducted between the total locus of control score and the total BSQ score. The results are presented in Table 7.

The results showed a statistically significant positive correlation between locus of control and body image concerns. This indicates that participants with a more external locus of control tended to report higher levels of concern or dissatisfaction regarding body shape. The strength of the association was weak to moderate.

**Figure 2:**
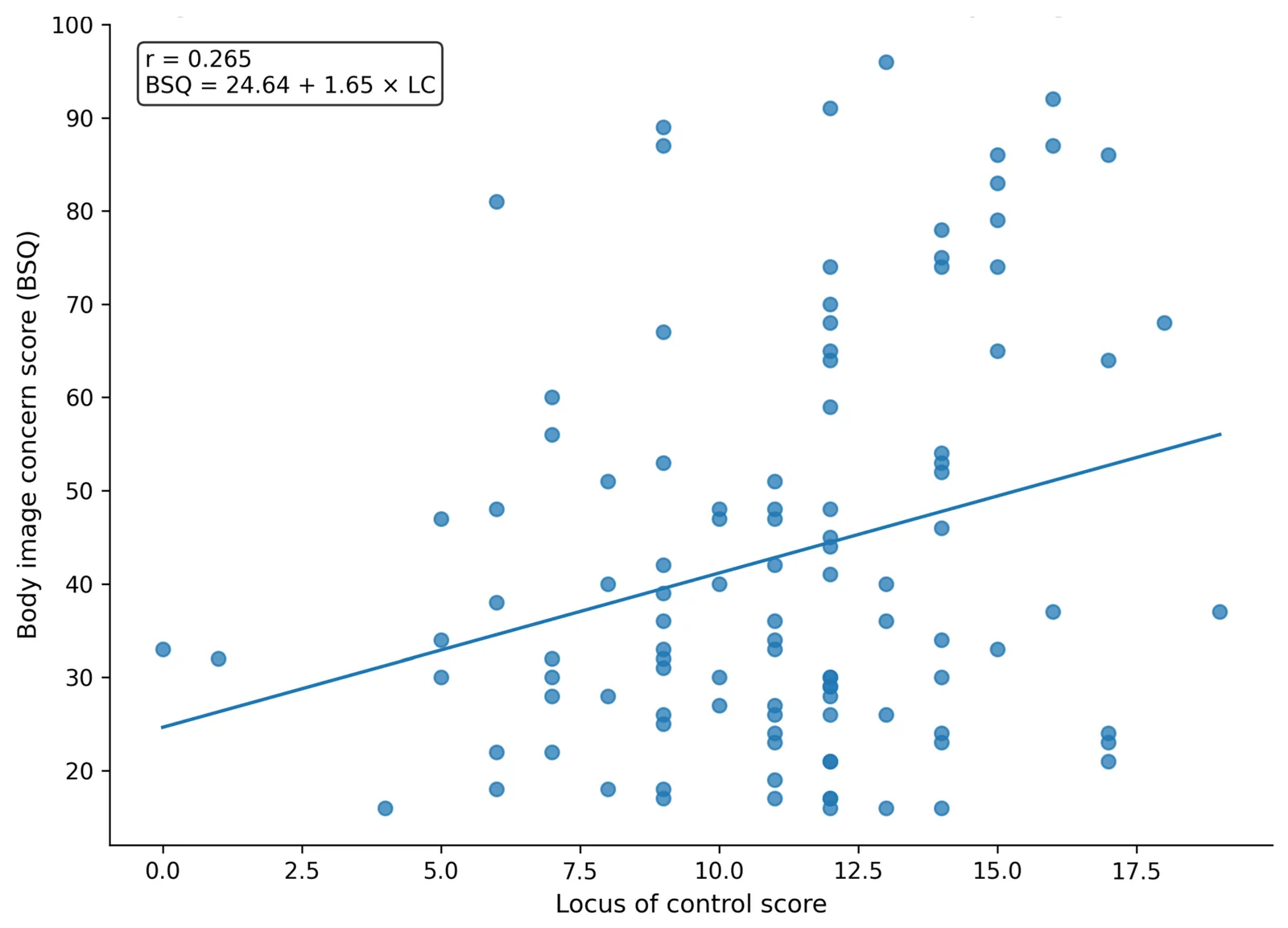
Scatterplot showing the association between locus of control and body image concerns, with the fitted linear regression line. The estimated regression equation was BSQ = 24.64 + 1.65 × locus of control, r = 0.265, *p* = 0.006, and *R*^2^ = 0.070. Higher locus-of-control scores indicate a more external locus of control, whereas higher BSQ scores indicate greater body shape concerns or dissatisfaction.

To illustrate the association between locus of control and body image concerns, Figure 2 presents a scatterplot of the two variables. The figure shows a positive relationship, indicating that higher scores on external locus of control tend to be associated with higher levels of body image concern.

After establishing a significant association between locus of control and body image concerns, a simple linear regression analysis was conducted. Locus of control was entered as the predictor variable, and body image concerns were entered as the dependent variable. The results are presented in Table 8.

The regression model was statistically significant, indicating that locus of control explained a significant proportion of variance in body image concerns. The positive regression coefficient showed that a more external locus of control was associated with higher body image concern scores. However, the proportion of explained variance was small, indicating that locus of control accounted for only a limited part of individual differences in body image concerns.

Therefore, the hypothesis stating that locus of control is associated with body image concerns and explains variance in body image concerns was supported.

Secondary analyses were conducted using the continuous EDDS-derived symptom-component scores in order to retain the full variation in self-reported symptoms. Spearman correlations and separate multiple regression models were calculated for the anorexia-related, binge-eating-related, and bulimia-related scores. The results are presented in Table 9.

Body image concerns were strongly and positively correlated with all three continuous symptom-component scores. The correlations were ρ = 0.822 for the anorexia-related score, ρ = 0.674 for the binge-eating-related score, and ρ = 0.621 for the bulimia-related score; all three associations remained significant after Holm correction, adjusted *p* < 0.001. Lower perceived social support was associated with a higher bulimia-related score, ρ = −0.317, Holm-adjusted *p* = 0.005. The association between lower social support and the binge-eating-related score was significant before, but not after, Holm correction, ρ = −0.223, unadjusted *p* = 0.020, adjusted *p* = 0.081. The anorexia-related score was not significantly correlated with perceived social support. None of the locus-of-control correlations remained significant after Holm correction, although the correlation with the anorexia-related score approached the adjusted threshold, ρ = 0.240, unadjusted *p* = 0.012, adjusted *p* = 0.061.

All three multiple regression models were statistically significant. The models explained 71.4% of the variance in the anorexia-related score (adjusted R^2^ = 0.706, robust *F*(3, 104) = 123.40, *p* < 0.001); 55.8% of the variance in the binge-eating-related score (adjusted R^2^ = 0.545, robust *F*(3, 104) = 46.61, *p* < 0.001); and 44.1% of the variance in the bulimia-related score (adjusted R^2^ = 0.425, robust *F*(3, 104) = 22.15, *p* < 0.001).

Body image concerns independently predicted all three symptom-component scores, with standardized coefficients of β = 0.834 for anorexia-related symptoms, β = 0.721 for binge-eating-related symptoms, and β = 0.592 for bulimia-related symptoms; all Holm-adjusted *p* values were <0.001. Lower perceived social support independently predicted higher binge-eating-related scores (β = −0.187, adjusted *p* = 0.043) and higher bulimia-related scores (β = −0.284, adjusted *p* = 0.002). Locus of control was not an independent predictor in any of the three models.

The results of the statistical analyses provided partial support for the hypotheses of the study. A summary of the hypothesis testing is presented in Table 10.

Overall, the findings showed that body image concerns were consistently higher among participants presenting eating-disorder tendencies. Perceived social support was significantly lower among participants with binge-eating and bulimic tendencies, but not among those with anorexic tendencies. At the uncorrected level, locus of control differed between participants with and without anorexic tendencies; however, the difference did not remain significant after Bonferroni correction. After correction, locus of control differed significantly only in the exploratory bulimia comparison, while no significant difference was found for binge-eating tendencies. Additionally, locus of control was significantly and positively associated with body image concerns and explained a small but statistically significant proportion of variance in body image concerns.

Exploratory Welch’s one-way analyses were conducted to examine whether perceived social support, locus of control, body image concerns, and body mass index differed among the four age categories (Table 11).

No statistically significant age-group differences were found in perceived social support, F(3, 35.83) = 0.12, *p* = 0.947, η^2^ = 0.004; locus of control, F(3, 41.52) = 0.90, *p* = 0.450, η^2^ = 0.021; or body image concerns, F(3, 35.18) = 0.82, *p* = 0.493, η^2^ = 0.022. An uncorrected difference was observed for BMI, F(3, 32.86) = 3.21, *p* = 0.036, η^2^ = 0.088; however, this result did not remain statistically significant after application of the Bonferroni-adjusted threshold of *p* < 0.0125.

Exploratory Welch’s independent-samples tests were conducted to compare male and female participants (Table 12).

No statistically significant gender differences were found in perceived social support (t(18.31) = 1.24, *p* = 0.231, Hedges’ g = 0.32), locus of control (t(17.62) = 1.16, *p* = 0.262, Hedges’ g = 0.32), or BMI (t(16.25) = −0.72, *p* = 0.483, Hedges’ g = −0.22). Female participants reported higher body image concern scores than male participants (M = 44.62 and M = 32.07, respectively, t(17.86) = 2.17, *p* = 0.044, Hedges’ g = 0.58). However, this difference did not remain statistically significant after application of the Bonferroni-adjusted threshold of *p* < 0.0125.

## 4. Discussion

The present study examined differences in perceived social support, locus of control, and body image concerns according to the presence or absence of subclinical eating-disorder tendencies. Specifically, the study focused on anorexic tendencies, binge-eating tendencies, and bulimic tendencies. In addition, the study examined the association between locus of control and body image concerns. Overall, the findings partially supported the working hypotheses. The most consistent result was observed for body image concerns, which were higher among participants presenting eating-disorder tendencies across all three comparison groups. Perceived social support and locus of control showed a more differentiated pattern, depending on the specific eating-disorder tendency examined.

The supplementary continuous-score analyses largely converged with the group comparisons and further identified body image concerns as the most consistent independent correlate of anorexia-related, binge-eating-related, and bulimia-related symptom variation.

Regarding the first hypothesis, the results showed that participants with anorexic tendencies reported significantly higher body image concerns than participants without anorexic tendencies. This finding supports the hypothesis and is consistent with previous research showing that body image disturbance is a central component of eating-disorder psychopathology. Prnjak et al., in a systematic review of body image facets in eating disorders, emphasized that body image disturbance is core to eating-disorder psychopathology [4]. Similarly, Abdoli et al. highlighted the strong links between body image disturbance, eating-disorder symptoms, self-esteem, and emotion regulation [42]. Therefore, the present finding suggests that even at a subclinical level, anorexic tendencies are accompanied by increased concern and dissatisfaction regarding body shape.

The strong difference in body image concerns between participants with and without anorexic tendencies can also be interpreted in relation to the cognitive and affective features of restrictive eating pathology. In anorexia-related symptomatology, body weight and shape are frequently overvalued and become central to self-evaluation. Although the present study did not include clinically diagnosed participants, the results suggest that body image concerns may already be elevated in individuals presenting subclinical anorexic tendencies. This is relevant from a prevention perspective, because subclinical manifestations may represent early psychological vulnerability before the development of a full clinical disorder.

The comparison involving anorexic tendencies also showed a difference in locus of control at the uncorrected level, with participants presenting anorexic tendencies reporting a more external locus of control. However, after applying Bonferroni correction, this result no longer reached the corrected significance threshold. Therefore, this finding should be interpreted cautiously. From a psychological perspective, the initial pattern may suggest that individuals with anorexic tendencies perceive life outcomes as being influenced more by external factors than by their own actions. This interpretation is compatible with research suggesting that control-related beliefs are relevant in eating-disorder symptoms. Di Corrado et al. found that intrapersonal variables, including self-efficacy and locus of control, were related to body image in a path-analysis model [24]. Froreich et al. also emphasized that control-related dimensions, especially feelings of ineffectiveness and fear of losing self-control, are important in disordered eating [25]. Nevertheless, because the corrected result was no longer significant, the present finding should be considered weak evidence rather than a robust confirmation of the hypothesis.

The first hypothesis was not supported with regard to perceived social support. Participants with anorexic tendencies did not differ significantly from participants without anorexic tendencies in perceived social support. This finding is only partially consistent with previous studies. Kim et al. found deficits in subjective social support across eating-disorder diagnostic groups [20], while Stern et al. showed prospective reciprocal relations between perceived peer support and eating-disorder symptoms in adolescent girls [22]. One possible explanation for the non-significant result in the present study is that the sample was nonclinical and the participants were classified according to subclinical tendencies rather than formal clinical diagnoses. Another explanation may be that the total perceived social support score did not distinguish between different sources of support, such as family, peers, romantic partners, or significant others. Stern, for example, found that perceived peer support, but not parental support, prospectively predicted later eating-disorder symptoms [22]. This suggests that future studies should examine different types and sources of social support separately.

Regarding the second hypothesis, participants with binge-eating tendencies reported significantly lower perceived social support than participants without binge-eating tendencies. This finding remained significant after Bonferroni correction and showed a meaningful effect size. The result is consistent with research showing that social and interpersonal factors are relevant in eating-disorder symptoms. Kim et al. reported deficits in subjective social support across eating-disorder diagnostic groups [20], and Makri et al. found that patients with eating disorders reported higher loneliness and lower perceived social support than healthy controls [21]. Similarly, other researchers described prospective associations between loneliness and disordered eating, suggesting that interpersonal difficulties may contribute to eating-related vulnerability. Psychiatric vulnerability may also involve psychological and biological processes beyond the variables examined in the present study. Dissociative mechanisms have been described as stress-related coping processes in severe psychopathology, although this evidence derives from dissociative identity disorder and cannot be generalized directly to subclinical eating-disorder tendencies [23,43]. The present finding is consistent with broader evidence linking loneliness and lower perceived support to eating-disorder symptoms [20,21,23]. However, the current cross-sectional analysis cannot determine whether reduced support contributes to binge-eating tendencies through distress-related coping or whether eating-related difficulties reduce perceived social connectedness.

The second hypothesis was also supported with regard to body image concerns. Participants with binge-eating tendencies reported significantly higher body image concerns than participants without binge-eating tendencies, and the effect size was large. This finding is consistent with the literature showing that body image disturbance is not limited to restrictive eating disorders but is also relevant in binge-eating symptoms. Lewer et al. emphasized that individuals with binge-eating disorder frequently present cognitive-affective body image disturbances [8]. Bianchi et al. also found that the perceived impact of body image on quality of life was associated with adolescents’ binge eating, partly through body image coping strategies [9]. The present finding therefore supports the idea that body shape concern is a transdiagnostic factor across different forms of eating-disorder vulnerability.

However, the second hypothesis was not supported for locus of control. No statistically significant difference was found between participants with and without binge-eating tendencies. This suggests that, in the present sample, binge-eating tendencies were more strongly related to perceived social support and body image concerns than to general locus of control. One possible explanation is that binge eating may be more closely connected to emotional regulation, loneliness, interpersonal distress, impulsivity, and eating-specific self-control than to broad beliefs about internal versus external control. Another possibility is that Rotter’s Locus of Control Scale may not be sufficiently specific to detect control-related processes involved in binge eating. Future research may benefit from using eating-specific measures, such as eating self-efficacy, loss-of-control eating measures, or health-related locus of control.

The exploratory analyses involving bulimic tendencies indicated significant differences in perceived social support, locus of control, and body image concerns. Participants with bulimic tendencies reported lower perceived social support, a more external locus of control, and higher body image concerns compared to participants without bulimic tendencies. These results are consistent with previous studies indicating that bulimic symptoms are associated with interpersonal difficulties, body dissatisfaction, and control-related processes. Kim et al. found that subjective social support deficits were present across eating-disorder groups and that objective social support was particularly reduced in bulimia nervosa [20]. The present findings are compatible with this pattern, as the bulimic-tendency group showed lower perceived social support than the non-bulimic group.

The strongest difference in the bulimia-related exploratory comparisons was observed for body image concerns. This was theoretically expected, because body dissatisfaction and overvaluation of shape and weight are central to bulimic symptomatology. Prnjak and Abdoli both emphasize the centrality of body image disturbance in eating-disorder symptoms [4,42]. In addition, the significant difference in locus of control suggests that participants with bulimic tendencies may experience a stronger perception that outcomes are externally determined. This may be relevant in the context of cycles of loss-of-control overeating followed by compensatory behaviors. However, because the group with bulimic tendencies was small and unequal in size compared to the non-bulimic group, these findings should be interpreted as exploratory and preliminary, even though they remained significant after Bonferroni correction.

The correlation analysis showed a significant positive association between external locus of control and body image concerns. This indicates that participants who reported a more external locus of control also tended to report higher concern or dissatisfaction regarding body shape. The regression analysis further showed that locus of control explained a statistically significant but small proportion of variance in body image concerns. This result supports the hypothesis that locus of control is related to body image concerns, although the magnitude of the association was limited. Di Corrado found that internal locus of control and self-efficacy were relevant intrapersonal factors in relation to body image [24]. Similarly, Froreich highlighted the importance of perceived control and ineffectiveness in disordered eating [25]. The present result is therefore compatible with previous research, but it also suggests that locus of control is only one of several factors associated with body image concerns.

The continuous-score analyses provided complementary evidence by retaining symptom variation that was not represented in the binary classifications. Body image concerns were strongly related to all three continuous symptom-component scores and remained the dominant predictor when perceived social support and locus of control were considered simultaneously. This convergence between the dichotomous and continuous analyses strengthens the interpretation of body image concerns as the most consistent correlate examined in the present study. Lower perceived social support independently predicted the binge-eating-related and bulimia-related scores, which was also consistent with the corresponding group comparisons. In contrast, general locus of control did not independently predict any of the three continuous symptom scores. Thus, the significant locus-of-control difference observed in the exploratory binary bulimia comparison was not reproduced as an independent association across the full range of bulimia-related scores. This difference may reflect the operational threshold used to define the bulimic-tendency group, shared variance among the psychosocial predictors, or measurement limitations of the general locus-of-control and bulimia-related scores.

The small positive coefficient for perceived social support in the anorexia-related regression should be interpreted cautiously. Perceived social support had an essentially null bivariate association with the anorexia-related score, and the positive coefficient emerged only after body image concerns and locus of control were entered simultaneously. This pattern suggests statistical suppression rather than a straightforward positive relationship and requires replication before substantive interpretation.

The small proportion of explained variance indicates that body image concerns are likely influenced by multiple psychological, interpersonal, and sociocultural factors. Recent reviews suggest that body dissatisfaction and eating-disorder symptoms are related to factors such as low self-esteem, emotion regulation difficulties, depression, anxiety, perfectionism, weight stigma, and media exposure. Biological stress-related pathways may likewise be relevant to broader psychiatric vulnerability; inflammatory markers have been investigated in schizophrenia and acute psychotic disorder within a vulnerability–stress–inflammation framework, but their relevance to subclinical eating-disorder tendencies remains uncertain [1,42,44]. Social media use has also been associated with body image concerns and disordered eating, particularly among adolescents [45,46]. Therefore, although locus of control was significantly associated with body image concerns in the present study, it should not be interpreted as the only or primary explanatory factor.

The exploratory age-group analyses indicated that perceived social support, locus of control, and body image concerns were relatively similar across the four age categories. The small effect sizes suggest that broad age-group membership was not strongly associated with the psychological variables in the present sample. The exploratory analyses did not identify corrected age-group differences in the measured outcomes. However, because age was not included as a covariate in the primary group comparisons, these analyses do not rule out age-related confounding. BMI showed a more noticeable age-related pattern, with the highest mean observed in the 28–35 years group and the lowest mean in 19–24 years group. However, this result did not remain statistically significant after correction for multiple comparisons, and should therefore be regarded as a preliminary tendency rather than evidence of a reliable age effect. Interpretation is also limited by unequal age-group sizes, particularly the small number of participants in the 18–19 years group, and by the use of broad categorical age groups rather than age as a continuous variable.

The exploratory gender analyses did not identify statistically reliable differences in perceived social support, locus of control, or BMI. Female participants reported higher body image concern scores than male participants, and the standardized difference was moderate. Nevertheless, this finding did not remain statistically significant after correction for multiple comparisons and should therefore be interpreted cautiously. The observed direction may be compatible with greater appearance-related concerns among women, but the current study cannot establish a stable gender difference. The sample included only 14 male participants and 94 female participants, producing substantial imbalance and limited precision for the male-group estimates. Accordingly, the nonsignificant findings should not be interpreted as demonstrating equivalence between male and female participants, and the nominal body image difference requires replication in a larger and more gender-balanced sample.

Taken together, the binary and continuous analyses suggest that body image concerns represent the most robust psychological correlate of subclinical eating-disorder tendencies in the present sample. Perceived social support appeared especially relevant to binge-eating-related and bulimia-related symptoms. Locus of control showed a more limited pattern: it was associated with body image concerns in the total sample and differed in the exploratory binary bulimia comparison, but it did not independently predict any of the continuous symptom-component scores. The three psychological variables therefore did not show equivalent or uniform associations across symptom dimensions.

The present findings extend the longstanding literature on body image and eating-disordered behavior in nonclinical populations. Their principal contribution lies in examining body image concerns, perceived social support, and locus of control within a single Romanian sample and in comparing their patterns across several eating-disorder tendency groups. The results do not indicate that body image concerns or subclinical eating symptoms are newly recognized phenomena. Instead, they show that established associations are also observable in this regional sample and that perceived social support and locus of control may differ in relevance across symptom profiles. From a prevention perspective, body image concerns and perceived social support may be considered candidate domains for further screening and intervention research. However, because the present study was cross-sectional and did not evaluate a screening program or intervention, it cannot establish that targeting these variables would improve clinical or preventive outcomes. The findings provide some indication of how the present Romania-based sample relates to the literature generated predominantly in other European and North American settings. The consistently higher body image concern scores across all three eating-disorder tendency profiles correspond closely with the broader literature and with Romanian studies documenting clinically relevant eating-disorder risk, body dissatisfaction, and appearance-related pressures. The direction of the body image findings is consistent with international reviews [4,43] and with Romanian studies involving adolescents, university students, and specific occupational groups [34,35,36]. However, the present convenience sample and the absence of a non-Romanian comparison group preclude conclusions about cross-cultural generalizability or uniquely Romanian effects. In contrast, perceived social support and locus of control showed less uniform profile-specific patterns. These results could reflect variation in interpersonal or control-related processes, but they should not be interpreted as specifically Romanian cultural effects. The study did not include a non-Romanian comparison group or direct measures of cultural values, appearance-ideal internalization, family pressure, media exposure, or culturally specific social-support expectations. Consequently, the findings provide Romanian contextual evidence but cannot determine whether the strength of these associations differs cross-nationally. A Romanian study of medical students found that perceived family pressure to be thin and weight- or appearance-related ridicule were associated with elevated eating-disorder risk; these variables were not measured in the present study [47].

### Study Limitations

Several limitations should be considered when interpreting the findings. First, the study used a cross-sectional design, which does not allow conclusions about causality or temporal direction. Although regression analyses were used, all findings should be understood as cross-sectional statistical associations rather than evidence that the psychosocial variables cause eating-disorder symptoms, that eating-disorder symptoms cause changes in these variables, or that locus of control causes body image concerns. Second, the sample was predominantly female, which limits the generalizability of the findings to male participants or gender-diverse populations. Third, the study relied on self-report questionnaires, which may be influenced by social desirability, response bias, or inaccurate self-perception. Fourth, no formal a priori power analysis was conducted, and the sample size was determined through convenience recruitment. Because the tendency classifications were assigned after the self-report data were scored, only 17 participants met the bulimic-tendency criterion. The corresponding comparisons had adequate sensitivity primarily for large effects and limited sensitivity for small or moderate effects. The unequal group sizes also reduced the precision of the estimates, as reflected in the confidence intervals. Consequently, the bulimia-related findings should be regarded as exploratory and require replication in larger samples containing more participants with elevated bulimic tendencies. Fifth, the study used broad total scores, particularly for perceived social support and locus of control. The study was not designed as a cross-cultural comparison: cultural identity, ethnicity, migration history, exposure to Western or globalized appearance ideals, family appearance-related pressure, and social media exposure were not assessed. Moreover, measurement invariance between Romanian and other national samples was not examined. Therefore, the results cannot establish that the observed associations are stronger, weaker, or qualitatively different in Romanian adults than in populations represented more frequently in the literature. Future research should distinguish between different sources of social support and use more specific measures of eating-related control, self-efficacy, or emotion regulation.

The eating-disorder tendency classifications were derived from self-reported questionnaire responses and were not verified through clinical interviews or medical examinations. They should therefore be interpreted as study-specific screening indicators of elevated symptom tendencies rather than diagnostic classifications. In addition, the results may be influenced by recall error, social desirability, inaccurate self-perception, and, where applicable, inaccuracies in self-reported height and weight.

Internal consistency was high for perceived social support, body image concerns, and the anorexia- and binge-eating-related continuous scores. However, the locus-of-control score showed modest internal consistency, and the three-item bulimia-related continuous score showed low internal consistency. The locus-of-control and bulimia-related findings may therefore be affected by measurement error and should be interpreted cautiously. In addition, the reliability coefficients apply to the continuous scores and do not establish the reliability or diagnostic validity of the study-specific binary tendency classifications.

Future studies should use larger and more balanced samples, including a higher number of male participants and participants from diverse age groups. Longitudinal designs would be especially useful to clarify whether perceived social support, locus of control, and body image concerns predict later increases in eating-disorder symptoms or whether eating-disorder symptoms lead to changes in these psychological variables. Future research should also examine additional variables, such as self-esteem, anxiety, depression, perfectionism, emotion regulation, body mass index, weight stigma, and social media exposure. These factors may help explain why body image concerns are strongly associated with eating-disorder tendencies and why perceived social support and locus of control show different patterns across symptom profiles. Also, future studies could examine whether broader psychopathological processes, including dissociative coping and stress-related inflammatory pathways, are associated with subclinical eating-disorder tendencies. Existing evidence for these processes has largely been obtained in dissociative and psychotic disorders, so their relevance to eating-related vulnerability should be investigated directly rather than assumed.

## 5. Conclusions

Body image concerns showed the most consistent associations across both the binary tendency classifications and the continuous symptom-component scores. Lower perceived social support was particularly relevant to binge-eating-related and bulimia-related symptoms. General locus of control was associated with body image concerns but did not independently predict continuous eating-disorder symptom scores. These findings support the importance of body image concerns and interpersonal resources in subclinical eating-disorder vulnerability, while the bulimia-related findings remain exploratory because of measurement limitations.

## Figures and Tables

**Table 1:** Demographic characteristics of the participants.

Variable	Category	*n*	%
Gender	Male	14	13.0
	Female	94	87.0
Area of residence	Urban	58	53.7
	Rural	50	46.3
Occupation	Pupil	5	4.6
	Student	18	16.7
	Student and employed	22	20.4
	Employed	58	53.7
	Without stable employment	5	4.6
Educational level	Middle school	5	4.6
	High school	16	14.8
	Bachelor’s degree	56	51.9
	Master’s degree	28	25.9
	Postgraduate study	3	2.8

Note: *n* = 108.

**Table 2:** Descriptive statistics for the main variables.

Variable	*n*	M	SD	Variance	Skewness	Kurtosis	Min.	Max.	Cronbach’s α
Perceived social support	108	83.33	9.79	95.89	−0.93	−0.16	59	96	0.92
Body image concerns	108	42.99	21.76	473.71	0.81	−0.41	16	96	0.96
Locus of control	108	11.11	3.50	12.23	−0.43	0.51	0	19	0.61
Anorexic tendencies	108	8.68	5.38	28.97	0.22	−1.04	0	18	0.86
Bulimic tendencies	108	2.71	3.02	9.12	1.43	1.69	0	12	0.46
Binge-eating tendencies	108	5.12	4.11	16.91	0.73	−0.75	1	14	0.84

Note. M = mean; SD = standard deviation; Min. = minimum value; Max. = maximum value. Internal-consistency coefficients refer to the continuous summed scores. For the dichotomously scored locus-of-control items, the reported coefficient is equivalent to KR-20. The coefficients do not apply to the binary eating-disorder tendency classifications.

**Table 3:** Distribution of participants by eating-disorder tendency group.

Eating-Disorder Tendency	Group	*n*	%
Anorexic tendencies	Without tendencies	66	61.1
	With tendencies	42	38.9
Bulimic tendencies	Without tendencies	91	84.3
	With tendencies	17	15.7
Binge-eating tendencies	Without tendencies	74	68.5
	With tendencies	34	31.5

Note: *n* = 108. Anorexia-related tendencies were defined as EDDS Items 2–4 ≥ 12 or BMI < 17.5 kg/m^2^. Bulimia-related tendencies were defined as Items 5 + 6 + 8 ≥ 4, compensatory-behavior Items 15–18 ≥ 8, and Item 3 or 4 ≥ 4. Binge-eating-related tendencies were defined as Items 5 + 6 + 7 ≥ 4. These classifications were based on self-report data, were calculated independently, could overlap, and do not represent clinical diagnoses.

**Table 4:** Comparison of perceived social support, locus of control, and body image concerns according to anorexic tendencies.

Variable	Group	*n*	M	SD	Test	t	df	*p*	Hedges’ g	95% CI for g	Interpretation
Perceived social support	Without anorexic tendencies	66	83.00	9.10	Student’s *t*-test	−0.44	106	0.660	−0.09	[−0.47, 0.30]	Not significant
	With anorexic tendencies	42	83.86	10.89							
Locus of control	Without anorexic tendencies	66	10.56	3.41	Student’s *t*-test	−2.08	106	0.040	−0.41	[−0.80, −0.02]	Significant (not significant after Bonferroni correction)
	With anorexic tendencies	42	11.98	3.50							
Body image concerns	Without anorexic tendencies	66	32.26	12.16	Welch’s *t*-test	−7.19	55.90	<0.001	−1.60	[−2.04, −1.16]	Significant
	With anorexic tendencies	42	59.86	22.91							

Note. Student’s *t*-tests were used for perceived social support and locus of control because the corresponding Levene tests were nonsignificant. Welch’s *t*-test was used for body image concerns because Levene’s test was significant.

**Table 5:** Comparison of perceived social support, locus of control, and body image concerns according to binge-eating tendencies.

Variable	Group	*n*	M	SD	Test	t	df	*p*	Hedges’ g	95% CI for g	Interpretation
Perceived social support	Without binge-eating tendencies	74	85.23	8.82	Welch *t*-test	2.88	54.63	0.006	0.63	[0.22, 1.05]	Significant
	With binge-eating tendencies	34	79.21	10.65							
Locus of control	Without binge-eating tendencies	74	10.82	3.31	Student *t*-test	−1.26	106	0.210	−0.26	[−0.66, 0.15]	Not significant
	With binge-eating tendencies	34	11.74	3.86							
Body image concerns	Without binge-eating tendencies	74	34.54	15.88	Welch *t*-test	−6.47	49.94	<0.001	−1.49	[−1.94, −1.04]	Significant
	With binge-eating tendencies	34	61.38	21.66							

Note. Hedges’ g was calculated using the difference between the group without binge-eating tendencies and the group with binge-eating tendencies. Positive values indicate higher scores in the group without binge-eating tendencies, whereas negative values indicate higher scores in the group with binge-eating tendencies. Welch’s *t*-test was reported for perceived social support and body image concerns because the assumption of homogeneity of variance was not met. Higher BSQ scores indicate greater concern or dissatisfaction regarding body shape. Higher locus-of-control scores indicates a more external locus of control. If Bonferroni correction is applied for the three binge-eating-related comparisons, the adjusted significance threshold is *p* < 0.017; under this correction, perceived social support and body image concerns remain statistically significant.

**Table 6:** Exploratory comparison of perceived social support, locus of control, and body image concerns according to bulimic tendencies.

Variable	Group	*n*	M	SD	Welch t	df	*p*	Hedges’ g	95% CI for g	Interpretation
Perceived social support	Without bulimic tendencies	91	84.49	9.17	2.62	20.44	0.016	0.77	[0.25, 1.30]	Significant, exploratory (borderline after Bonferroni correction)
	With bulimic tendencies	17	77.12	10.91						
Locus of control	Without bulimic tendencies	91	10.73	3.47	−3.08	25.26	0.005	−0.72	[−1.24, −0.19]	Significant, exploratory
	With bulimic tendencies	17	13.18	2.92						
Body image concerns	Without bulimic tendencies	91	37.60	17.92	−7.31	22.58	<0.001	−1.90	[−2.48, −1.32]	Significant, exploratory
	With bulimic tendencies	17	71.82	17.68						

Note. Welch’s *t*-test was used for all bulimia-related comparisons because of the marked group-size imbalance, although Levene’s tests were nonsignificant for all three outcomes. Because only 17 participants met the bulimic-tendency criterion, these comparisons had limited statistical sensitivity for small and moderate effects. The estimates and confidence intervals should therefore be interpreted as exploratory.

**Table 7:** Correlation between locus of control and body image concerns.

Variables	*n*	r	*p*	Interpretation
Locus of control and body image concerns	108	0.265	0.006	Significant positive correlation

Note. Higher scores on locus of control indicate a more external locus of control. Higher scores on body image concerns indicate greater concern or dissatisfaction regarding body shape.

**Table 8:** Simple linear regression predicting body image concerns from locus of control.

Predictor	B	SE B	β	t	*p*
Constant	24.64	6.79	—	3.63	<0.001
Locus of control	1.65	0.58	0.265	2.83	0.006

Model summary: R = 0.265, R^2^ = 0.070, adjusted R^2^ = 0.062, *F*(1, 106) = 8.03, *p* = 0.006. Note. The dependent variable was body image concerns. Higher scores on body image concerns indicate greater concern or dissatisfaction regarding body shape. Higher locus of control scores indicates a more external locus of control.

**Table 9:** Associations of continuous eating-disorder symptom-component scores with perceived social support, locus of control, and body image concerns.

**Panel (A): Spearman rank-order correlations**												
**Continuous Symptom Score**		**Psychosocial** **Variable**		**Spearman Rho**		**95% Bootstrap CI**		***p***	**Holm-Adjusted *p***		**Holm Result**	
**Anorexia-related score**		Perceived social support		0.065		[−0.131, 0.253]		0.504	0.504		Not significant	
		Locus of control		0.240		[0.037, 0.422]		0.012	0.061		Not significant	
		Body image concerns		0.822		[0.734, 0.883]		<0.001	<0.001		Significant	
**Binge-eating-related score**		Perceived social support		−0.223		[−0.406, −0.021]		0.020	0.081		Not significant	
		Locus of control		0.161		[−0.018, 0.330]		0.097	0.260		Not significant	
		Body image concerns		0.674		[0.541, 0.770]		<0.001	<0.001		Significant	
**Bulimia-related score**		Perceived social support		−0.317		[−0.482, −0.125]		<0.001	0.005		Significant	
		Locus of control		0.166		[−0.024, 0.339]		0.087	0.260		Not significant	
		Body image concerns		0.621		[0.480, 0.723]		<0.001	<0.001		Significant	
**Panel (B): Multiple linear regressions with HC3 robust standard errors**												
**Continuous Symptom Score**	**Predictor**		**B**	**HC3 SE**	**Standardized Beta**		**95% CI for B**		***p***	**Holm-Adjusted *p***		**VIF**
**Anorexia-related score**	Perceived social support		0.074	0.027	0.135		[0.021, 0.127]		0.007	0.035		1.03
	Locus of control		0.079	0.074	0.052		[−0.067, 0.226]		0.284	0.825		1.09
	Body image concerns		0.206	0.011	0.834		[0.184, 0.229]		<0.001	<0.001		1.08
**Binge-eating-related score**	Perceived social support		−0.078	0.030	−0.187		[−0.138, −0.019]		0.011	0.043		1.03
	Locus of control		−0.081	0.078	−0.069		[−0.235, 0.073]		0.298	0.825		1.09
	Body image concerns		0.136	0.013	0.721		[0.111, 0.161]		<0.001	<0.001		1.08
**Bulimia-related score**	Perceived social support		−0.088	0.024	−0.284		[−0.135, −0.040]		<0.001	0.002		1.03
	Locus of control		−0.069	0.063	−0.080		[−0.193, 0.056]		0.275	0.825		1.09
	Body image concerns		0.082	0.012	0.592		[0.058, 0.106]		<0.001	<0.001		1.08

**Table 10:** Summary of hypothesis testing.

Hypothesis	Description	Result	Conclusion
H1	Participants with anorexic tendencies will report greater body image concerns, lower perceived social support, and a more external locus of control than participants without such tendencies.	Body image concerns differed significantly. The locus-of-control difference was significant only before Bonferroni correction, and perceived social support did not differ significantly.	Partially supported
H2	Participants with binge-eating tendencies will report greater body image concerns, lower perceived social support, and a more external locus of control than participants without such tendencies.	Significant differences were found for perceived social support and body image concerns, but not for locus of control.	Partially supported
H3	Participants with bulimic tendencies will report greater body image concerns, lower perceived social support, and a more external locus of control than participants without such tendencies.	Exploratory analyses indicated significant differences for perceived social support, locus of control, and body image concerns.	Supported, but interpreted with caution
H4	A more external locus of control will be positively associated with body image concerns.	A significant positive correlation was found between locus of control and body image concerns.	Supported

**Table 11:** Descriptive and inferential results according to age group.

Outcome	Age Group Mean = 18.6 Years, *n* = 10	Age Group Mean = 22.7 Years, *n* = 44	Age Group Mean = 25.1 Years, *n* = 23	Age Group Mean = 33.6 Years, *n* = 31	Welch’s Test	*p*	η^2^
Perceived social support	84.00 ± 8.16	83.89 ± 9.58	82.65 ± 10.27	82.84 ± 10.58	F(3, 35.83) = 0.12	0.947	0.004
Locus of control	10.50 ± 1.84	11.48 ± 3.37	11.57 ± 3.41	10.45 ± 4.11	F(3, 41.52) = 0.90	0.450	0.021
Body image concerns	39.10 ± 19.91	45.30 ± 22.77	45.96 ± 23.00	38.77 ± 20.04	F(3, 35.18) = 0.82	0.493	0.022
BMI	23.52 ± 4.63	22.11 ± 3.34	23.68 ± 3.66	24.95 ± 4.40	F(3, 32.86) = 3.21	0.036	0.088

**Table 12:** Descriptive and inferential results according to gender.

Outcome	Male, *n* = 14	Female, *n* = 94	Female–Male Difference	Welch’s t	*p*	Hedges’ g
Perceived social support	80.57 ± 8.78	83.74 ± 9.91	+3.17	t(18.31) = 1.24	0.231	0.32
Locus of control	10.14 ± 3.32	11.26 ± 3.52	+1.11	t(17.62) = 1.16	0.262	0.32
Body image concerns	32.07 ± 19.99	44.62 ± 21.65	+12.55	t(17.86) = 2.17	0.044	0.58
BMI	24.17 ± 4.39	23.28 ± 3.93	−0.89	t(16.25) = −0.72	0.483	−0.22

## Data Availability

The original contributions presented in this study are included in the article. Further inquiries can be directed to the corresponding authors.

## References

[1] Barakat S., McLean S.A., Bryant E., Le A., Marks P., Aouad P., Barakat S., Boakes R., Brennan L., National Eating Disorder Research Consortium (2023). Risk Factors for Eating Disorders: Findings from a Rapid Review. J. Eat. Disord..

[2] Button E.J., Whitehouse A. (1981). Subclinical Anorexia Nervosa. Psychol. Med..

[3] Shisslak C.M., Crago M., Estes L.S. (1995). The Spectrum of Eating Disturbances. Int. J. Eat. Disord..

[4] Prnjak K., Jukic I., Mitchison D., Griffiths S., Hay P. (2022). Body Image as a Multidimensional Concept: A Systematic Review of Body Image Facets in Eating Disorders and Muscle Dysmorphia. Body Image.

[5] Williamson G., Osa M.L., Budd E., Kelly N.R. (2021). Weight-Related Teasing Is Associated with Body Concerns, Disordered Eating, and Health Diagnoses in Racially and Ethnically Diverse Young Men. Body Image.

[6] Aparicio-Martinez P., Perea-Moreno A.-J., Martinez-Jimenez M.P., Redel-Macías M.D., Pagliari C., Vaquero-Abellan M. (2019). Social Media, Thin-Ideal, Body Dissatisfaction and Disordered Eating Attitudes: An Exploratory Analysis. Int. J. Environ. Res. Public Health.

[7] Mallaram G.K., Sharma P., Kattula D., Singh S., Pavuluru P. (2023). Body Image Perception, Eating Disorder Behavior, Self-Esteem and Quality of Life: A Cross-Sectional Study among Female Medical Students. J. Eat. Disord..

[8] Lewer M., Bauer A., Hartmann A., Vocks S. (2017). Different Facets of Body Image Disturbance in Binge Eating Disorder: A Review. Nutrients.

[9] Bianchi D., Schinelli A., Fatta L.M., Lonigro A., Lucidi F., Laghi F. (2023). Body Image Impact on Quality of Life and Adolescents’ Binge Eating: The Indirect Role of Body Image Coping Strategies. Eat. Weight. Disord..

[10] Rodin J., Silberstein L., Striegel-Moore R. (1984). Women and Weight: A Normative Discontent. Neb. Symp. Motiv..

[11] Stice E., Schupak-Neuberg E., Shaw H.E., Stein R.I. (1994). Relation of Media Exposure to Eating Disorder Symptomatology: An Examination of Mediating Mechanisms. J. Abnorm. Psychol..

[12] Van Den Berg P., Thompson J.K., Obremski-Brandon K., Coovert M. (2002). The Tripartite Influence model of body image and eating disturbance: A covariance structure modeling investigation testing the mediational role of appearance comparison. J. Psychosom. Res..

[13] Groesz L.M., Levine M.P., Murnen S.K. (2002). The Effect of Experimental Presentation of Thin Media Images on Body Satisfaction: A Meta-analytic Review. Int. J. Eat. Disord..

[14] Perloff R.M. (2014). Social Media Effects on Young Women’s Body Image Concerns: Theoretical Perspectives and an Agenda for Research. Sex Roles.

[15] Holland G., Tiggemann M. (2016). A Systematic Review of the Impact of the Use of Social Networking Sites on Body Image and Disordered Eating Outcomes. Body Image.

[16] Griffiths S., Harris E.A., Whitehead G., Angelopoulos F., Stone B., Grey W., Dennis S. (2024). Does TikTok Contribute to Eating Disorders? A Comparison of the TikTok Algorithms Belonging to Individuals with Eating Disorders versus Healthy Controls. Body Image.

[17] Ma J., Wang K., Thompson J.K. (2023). Translation and Psychometric Properties of the Chinese Version of the Sociocultural Attitudes Towards Appearance Questionnaire-4 (SATAQ-4) in College Students. Body Image.

[18] Sanzari C.M., Gorrell S., Anderson L.M., Reilly E.E., Niemiec M.A., Orloff N.C., Anderson D.A., Hormes J.M. (2023). The Impact of Social Media Use on Body Image and Disordered Eating Behaviors: Content Matters More than Duration of Exposure. Eat. Behav..

[19] Leonidas C., Santos M. (2014). Social Support Networks and Eating Disorders: An Integrative Review of the Literature. Neuropsychiatr. Dis. Treat..

[20] Kim S., Smith K., Udo T., Mason T. (2023). Social Support across Eating Disorder Diagnostic Groups: Results from the National Epidemiologic Survey on Alcohol and Related Conditions-III (NESARC-III). Eat. Behav..

[21] Makri E., Michopoulos I., Gonidakis F. (2022). Investigation of Loneliness and Social Support in Patients with Eating Disorders: A Case-Control Study. Psychiatry Int..

[22] Stern M., Rubino L., Desjardins C., Stice E. (2023). Prospective Reciprocal Relations between Social Support and Eating Disorder Symptoms. J. Psychopathol. Clin. Sci..

[23] Cortés-García L., Rodríguez-Cano R., Von Soest T. (2022). Prospective Associations between Loneliness and Disordered Eating from Early Adolescence to Adulthood. Int. J. Eat. Disord..

[24] Di Corrado D., Coco M., Guarnera M., Maldonato N.M., Quartiroli A., Magnano P. (2021). The Influence of Self-Efficacy and Locus of Control on Body Image: A Path-Analysis in Aspiring Fashion Models, Athletes and Students. Int. J. Environ. Res. Public Health.

[25] Froreich F.V., Vartanian L.R., Grisham J.R., Touyz S.W. (2016). Dimensions of Control and Their Relation to Disordered Eating Behaviours and Obsessive-Compulsive Symptoms. J. Eat. Disord..

[26] Manaf N.A., Saravanan C., Zuhrah B. (2016). The Prevalence and Inter-Relationship of Negative Body Image Perception, Depression and Susceptibility to Eating Disorders among Female Medical Undergraduate Students. J. Clin. Diagn. Res..

[27] Lian Y., Liu S., Zhang D., Qiao D., Zhang Z., Mi G., Liu Z., Wu X. (2025). Emotion Dysregulation and Eating Disorder Symptoms: A Network Analysis in College Students with Subclinical Eating Disorders. J. Eat. Disord..

[28] Stice E. (2002). Risk and Maintenance Factors for Eating Pathology: A Meta-Analytic Review. Psychol. Bull..

[29] Ladner J., Lukács A., Brumboiu I., Ciobanu E., Croitoru C., Sasvári P., Tavolacci M.P. (2019). Eating Disorders among University Students: A Public Health Challenge. An European Study. Eur. J. Public Health.

[30] Kovács Krizbai T., Szabó P. (2009). [Prevalence of eating disorders in Romanian, Hungarian and Saxon secondary school students in Transylvania]. Psychiatr. Hung..

[31] Joja O., Von Wietersheim J. (2012). A Cross-Cultural Comparison between EDI Results of Romanian and German Students. Procedia Soc. Behav. Sci..

[32] King M. (1989). Locus of Control in Women with Eating Pathology. Psychol. Med..

[33] Ispas A.G., Forray A.I., Lacurezeanu A., Petreuș D., Gavrilaș L.I., Cherecheș R.M. (2025). Eating Disorder Risk Among Adolescents: The Influence of Dietary Patterns, Physical Activity, and BMI. Nutrients.

[34] Iorga M., Manole I., Pop L., Muraru I.-D., Petrariu F.-D. (2018). Eating Disorders in Relationship with Dietary Habits among Pharmacy Students in Romania. Pharmacy.

[35] Zancu S.A., Rodgers R.F., Enea V. (2019). Self-Determined Motivation for Eating Behavior Regulation and Sociocultural Influences among Romanian Fashion Models. Body Image.

[36] Cutrona C.E., Russel D.W. (1987). The Provisions of Social Relationships and Adaptation to Stress. Advances in Personal Relationships.

[37] Dughi T.S., Demeter E., Vancu G.S. (2020). Perceived Social Support and Anxiety: A Correlational Analysis. J. Plus Educ..

[38] Stice E., Telch C.F., Rizvi S.L. (2000). Development and Validation of the Eating Disorder Diagnostic Scale: A Brief Self-Report Measure of Anorexia, Bulimia, and Binge-Eating Disorder. Psychol. Assess..

[39] Cooper P.J., Taylor M.J., Cooper Z., Fairbum C.G. (1987). The Development and Validation of the Body Shape Questionnaire. Int. J. Eat. Disord..

[40] Evans C., Dolan B. (1993). Body Shape Questionnaire: Derivation of Shortened “Alternate Forms”. Int. J. Eat. Disord..

[41] Rotter J.B. (1966). Generalized Expectancies for Internal versus External Control of Reinforcement. Psychol. Monogr. Gen. Appl..

[42] Abdoli M., Schiechtl E., Rosato M.S., Mangweth-Matzek B., Cotrufo P., Hüfner K. (2025). Body Image, Self-Esteem, Emotion Regulation, and Eating Disorders in Adults: A Systematic Review. Neuropsychiatrie.

[43] Trifu S. (2019). Dissociative Identity Disorder. Psychotic Functioning and Impairment of Growing-up Processes. J. Educ. Sci. Psychol..

[44] Segarceanu L.-M., Zanfir A.-G., Minca D.G., Trifu S. (2025). Evaluation of Inflammatory Markers in Perception Disorders in Major Psychiatric Pathology. Int. J. Mol. Sci..

[45] Rodgers R.F., Slater A., Gordon C.S., McLean S.A., Jarman H.K., Paxton S.J. (2020). A Biopsychosocial Model of Social Media Use and Body Image Concerns, Disordered Eating, and Muscle-Building Behaviors among Adolescent Girls and Boys. J. Youth Adolesc..

[46] Suhag K., Rauniyar S. (2024). Social Media Effects Regarding Eating Disorders and Body Image in Young Adolescents. Cureus.

[47] Motorga R., Ionescu M., Nechita F., Micu D., Băluțoiu I., Dinu M.M., Nechita D. (2025). Eating Disorders in Medical Students: Prevalence, Risk Factors, Comparison with the General Population. Front. Psychol..

